# Supplementary material for: Obesity, Bone Loss, and Periodontitis: The Interlink
Source: Biomolecules. 2022 Jun 22;12(7):865. doi: 10.3390/biom12070865 (PMC9313439; doi:10.3390/biom12070865)
Supplement: Supplementary file 1 [file biomolecules-12-00865-s001.zip › biomolecules-1773739-SI.pdf]

biomolecules

Supplementary information

Obesity, Bone Loss, and Periodontitis: The Interlink

Pengfei Zhao [1], Aimin Xu [2] and Wai Keung Leung [1,*]

[1] Faculty of Dentistry, The University of Hong Kong, Hong Kong SAR, China; pfzhao@connect.hku.hk
[2] Department of Medicine, Li Ka Shing Faculty of Medicine, The University of Hong Kong, Hong Kong SAR, China; amxu@hku.hk
* Correspondence: ewkleung@hku.hk; Tel.: +852-2859-0417

Table S1. Obesity and periodontitis systematic reviews and meta-analyses search strategies.

Database (Start date)	Search strategies (Search end date: March 31, 2022)
PubMed (1946)	((((Obesity [MeSH Terms]) OR (obese)) OR (overweight)) AND (((Periodontitis [MeSH Terms]) OR (Chronic Periodontitis [MeSH Terms])) OR (periodontal disease))) AND (Systematic review OR review OR overview OR meta-analysis)
EMBASE (1974)	(exp “obesity”/ or exp “obese patient”/) AND (exp “periodontitis”/ or exp “chronic periodontitis”/ or exp “periodontal disease”/) AND (exp "review"/ or exp "systematic review"/ or exp “meta analysis”/ or overview.mp.)
Web of Science (1956)	((TS=(obesity)) OR TS=(obese)) AND (((TS=(periodontitis)) OR TS=(chronic periodontitis)) OR TS=(periodontal disease)) AND ((((TS=(review)) OR TS=(systematic review)) OR TS=(meta analysis)) OR TS=(overview))

Table S2. Assessment of methodological quality by Risk of Bias in Systematic Reviews (ROBIS) tool concerning systematic reviews and meta-analyses mentioned.

	Phase 1	Phase 2				Phase 3
**Authors, year**	**Assessing relevance**	**1. Study eligibility criteria**	**2. Identification and selection of studies**	**3. Data collection and study appraisal**	**4. Synthesis and findings**	**Risk of bias in the review**
Periodontitis and obesity						
Chaffee *et al.* 2010 [12]	Yes	Low	Low	Low	High[1]	High[1]
Suvan *et al.* 2011 [13]	Yes	Low	Low	Low	Unclear[2]	Unclear[2]
de Moura-Grec *et al.* 2014 [14]	Yes	Low	Low	High[c]	Unclear[2]	High[2, 3]
Keller *et al.* 2015 [20]	Yes	Low	Low	High[c]	High[4]	High[3, 4]
Li *et al.* 2015 [15]	Yes	Low	Low	Low	Low	Low
Nascimento *et al.* 2015 [16]	Yes	Low	Low	Low	Low	Low

Nascimento *et al*. 2016 [17]	Partial	Low	Low	Low	Low	Low
Martens *et al*. 2017 [18]	Yes	Low	Low	Low	High[5]	High[5]
Martinez-Herrera *et al*. 2017 [21]	Yes	Low	Low	High[3]	High[4]	High[3, 4]
Khan *et al*. 2018 [22]	Yes	Low	Low	Low	High[4]	High[4]
Foratori-Junior et al. 2022 [19]	Yes	Low	Low	Low	Low	Low
Periodontitis GCF cytokines and obesity						
Akram *et al*. 2015 [114]	Yes	Low	Low	Low	Low	Low
Exercise intervention adjunct periodontal therapy						
Gerber *et al*. 2016 [146]	Yes	Low	Low	Low	High[4]	High[4]
Ramseier *et al*. [154]	Yes	Low	Low	Low	High[4]	High[4]
Bone mineral density and obesity						
Turcotte *et al*. 2021 [5]	Yes	Low	Low	Low	Low	Low

[1] Heterogeneity of included studies is considerable with 13 of 28 primary studies rated with high risk of bias and were not addressed in the synthesis.
[2] Robustness of the findings is not secured because funnel plot or sensitivity analyses were not attempted.
[3] Risk of bias (or methodological quality) not assessed using appropriate criteria.
[4] ROBIS Domain 4.4, 4.5, and 4.6 were not addressed in the systematic review concern without meta-analysis (i.e. between study variation-heterogeneity, funnel plot/sensitivity analysis, bias — publication bias data not available).
[5] 6 of 7 included studies did not report periodontal disease definition, the synthesis may not appropriately reflect any similarity in outcomes while potential heterogeneity was considerable and not addressed.
